# Effects of Receiving Two Initial Feedings of Colostrum on the Average Daily Gain and Health of Pre-Weaning Group Housed Holstein Heifer Calves

**DOI:** 10.3390/ani11113209

**Published:** 2021-11-10

**Authors:** Qiu-Di Zheng, Francisco A. Leal Yepes

**Affiliations:** 1 Department of Population Medicine and Diagnostic Sciences, College of Veterinary Medicine Cornell University, Ithaca, NY 14853, USA; qdz2@cornell.edu; 2 Department of Veterinary Clinical Sciences, College of Veterinary Medicine, Washington State University, Pullman, WA 99164-6610, USA

**Keywords:** colostrum, dairy calf, passive immune transfer

## Abstract

**Simple Summary:**

High levels of bioactive factors in colostrum may benefit the growth of dairy calves when provided after the loss of macromolecule absorption ability at the gastrointestinal level. We explored the effects of feeding two feedings of colostrum instead of only one on dairy calves and analyzed the impact of growth and disease during the pre-weaning period. Calf rearing accounts for a significant expense on farms. Therefore, raising calves without additional costs becomes important economically and for improving animal welfare. Feeding calves with two feedings of colostrum did not significantly affect growth but increased total serum protein in those calves.

**Abstract:**

We studied the effect on average daily gain (ADG) and health of an additional colostrum feeding to Holstein dairy heifers 12–16 h after the first colostrum feeding, provided within 2 h of birth. Calves (n = 190) with an average birth weight of 38.8 kg (29.5–52.6 kg) were randomly enrolled in blocks to either the control (CON) or colostrum (COL). The CON received 3 L of acidified pasteurized whole milk, and the COL received 3 L of pasteurized colostrum [average: 25.5 (24.7–26.4)% Brix]. Calves were group-housed, weighed, withers height measured weekly. Serum was obtained and analyzed with a% Brix refractometer. Mixed linear models were used to assess the differences in ADG, body weight, and height between the treatment and control. There was no difference in ADG between the COL and CON. However, serum % Brix was higher in the COL group (9.7%) than in the CON group (9.2%). Calves in the COL had more antibiotic treatments for respiratory diseases but fewer antibiotic treatments for otitis than the CON. In conclusion, providing an extra feeding of colostrum did not contribute to ADG of Holstein heifers during the pre-weaning period but did provide them with a higher total serum protein concentration.

## 1. Introduction

Colostrum is the first milk secreted after parturition, and it has a greater concentration of antibodies that confers passive immunity to the neonatal calf [1]. In several studies, colostrum composition is found to be more intricate than just containing greater concentrations of immunoglobulins [2,3,4]. The abundance of colostrum components can be categorized into two main groups–the nutritive and the non-nutritive elements [5]. The nutritive components deliver adequate energy and building blocks to support calf growth and thermoregulation. The non-nutritive components include the immunoglobulins and bioactive factors such as insulin-like growth factors (IGFs), glucagon-like peptides, oligosaccharides, glucocorticoids, growth hormones, and leptin [6]. Some of these factors may affect gastrointestinal growth signaling pathways [7,8]. Fischer et al., (2018) [9] demonstrated that calves fed with colostrum with greater concentrations of oligosaccharides had a greater prevalence of beneficial bacteria in their gastrointestinal tracts. Moreover, Hammon et al., (1998) [10] observed an increased insulin response in calves that were fed colostrum instead of colostrum replacer. The blood IGF-1 concentrations in calves were negatively affected when colostrum intake was delayed or omitted [11].

The complex interactions of these bioactive factors are not yet entirely understood at the molecular level; previous studies elucidate how providing these bioactive factors in the initial diet may positively affect the overall morphology of small intestinal villi size and weight [12,13,14]. Additionally, some of these bioactive factors may reduce morbidity and mortality during the pre-weaning period in dairy calves [15,16]. The Animal and Plant Health Inspection Service U.S. Department Of Agriculture (2014) [17] showed that diarrhea and pneumonia are the two primary causes of mortality in pre-weaned dairy calves, at 56.4% and 24%, respectively.

The consequences of calf-hood diseases during the pre-weaning period may substantially impact their herd’s growth rates and longevity [18]. Previous reports have shown an increased milk production during the first lactation, approximately 704 kg for every additional kilogram of average daily gain (ADG) during the pre-weaning period [19]. Soberon et al., (2012) [20] have shown that calves treated for diarrhea produced 493 kg less milk during their first lactation than those without any diarrhea events. Besides, the top reasons for antimicrobial use in pre-weaned heifers in most U.S. dairy operations are treating sick calves for gastrointestinal problems and pneumonia, at 16% and 11.4%, respectively [17].

Feeding an adequate amount of good quality colostrum, IgG concentration > 50 g/L at 10–12% of the calf’s body weight, to newborn calves has been the mainstay of current practices on most dairy farms and the focus of colostrum research for the past three decades [3,15,21]. Based on the criteria, around 4 L of colostrum is fed within 4 h of the neonatal life to ensure adequate immunoglobulins delivery to the agammaglobulinaemic calf before a reduction of permeability to these large molecules occurs in the calf’s gastrointestinal tract [22]. Furthermore, in instances where colostrum intake is impeded by feeding method, i.e., nipple bottle versus esophageal tubing, a second feeding is recommended 6 h later, whereas esophageal tubing efficiently delivers 4 L independent of calf’s intake ability [15]. Following colostrum intake, calves are routinely fed various milk products in different amounts until weaning, depending on the on-farm availability of milk products. Thus, finding ways to improve calf health is undeniably crucial for maintaining dairy farms’ economic efficiency and animal welfare.

Our objective was to compare the benefits of providing calves with two feedings of colostrum 12–16 h apart to calves that get only one colostrum feeding, both feedings delivered via an esophageal tube. Specifically, to compare the differences in ADG, and the number of antibiotic treatments for neonatal diseases. Therefore, we hypothesized that calves fed with two feedings of colostrum within the first 12–16 h of life achieved greater weekly ADG. These calves will also have fewer disease incidences and require fewer treatments.

## 2. Materials and Methods

All animal procedures were approved by the Cornell University Institutional Animal Care and Use Committee (protocol no. 2019-0026). Holstein heifer calves (n = 190) from a family-owned commercial herd in Tompkins County, New York, USA, were enrolled from August 2019 to April 2020. The sample size of 95 animals per treatment was based on identifying a biological difference in average daily gain (ADG) between the control and the treatment of 0.07 kg with SD of 0.13 kg, confidence level of 95%, power 95%, and accounting for 10% of mortality. Heifer calves were blocked in pairs based on their birth date then randomly assigned to either treatment or control using PROC PLAN (SAS 9.4, SAS Institute Inc., Cary, NC, USA). The heifer calves enrolled were singletons born unassisted from dams with a gestation length (278 ± 8 days). Heifer calves weighing between 31.5 and 54 kg and without any phenotypical abnormalities at birth were included in the current study. All calves were removed from the dam immediately after birth, weighed using a scale, and navel dipped with 7% iodine.

Treatment calves (**COL**; n = 95) received two feedings of pooled maternal colostrum with an esophageal tube (Perfectudder bags system; Dairy Tech Inc. Greeley, CO, USA). The first feeding was 4 L within 2 h of age, and a second feeding of 3 L between 12–16 h of age. 12–16 h coincides with the farm movement of heifers from maternity pen to the calf barn and where they will be fed at the nipple. Control calves (**CON**; n = 95) received one feeding of 4 L of pooled colostrum within 2 h of age followed by 3 L of pasteurized acidified milk between 12–16 h of age with an esophageal tube (Perfectudder bags system). Pooled colostrum with a % Brix value ≥ 22 was collected using the 4 L and 3 L, heat-treated at 60 °C for 60 min. All the % Brix measurements were performed using a digital dairy refractometer (IgG colostrum and serum concentration—MISCO DD-2 Refractometer, Solon, OH, USA). Colostrum was stored at 4 °C for no longer than five days to ensure good colostrum quality and maintain an IgG concentration greater than 50 g/L delivered to each calf at the first feeding [15]. Milk fed to the control calves was collected similarly with 3 L Perfectudder bags. This second feeding milk used was pasteurized acidified milk, which was the same as those being fed to all calves at the farm facility. Prior to all feedings, the colostrum and acidified milk were thawed in a water bath maintained at a consistent temperature of 40 °C until the temperature of milk and colostrum reached a temperature of 40 °C, approximately 20 min. One of the authors (Zheng) performed all second feedings for the COL and CON calves, % Brix were measured in both COL and CON, and the farm personnel was blinded to all the treatments.

All enrolled calves were moved from the maternity area to an independently operated calf barn post-second feeding. Calves enrolled in the study were commingled with other calves on the farm that were not enrolled in our study. Calves enrolled from November 2019 to April 2020 were provided with calf blankets (Udder Tech, Rosemount, MN, USA) from 1 d of life. Calves were group-housed in a naturally ventilated barn supplemented with positive pressure ventilation. Approximately 20 calves within 1–2 days of age difference were housed in 13 × 6 m pens bedded with straw (3.91 m^2^/calf). All calves had ad libitum to pasteurized acidified milk delivered through a milk line with nipple bars, 6 nipples per pen; 3.33 calves/nipple. Milk was pasteurized at 72 °C for 15 s, then formic acid diluted with water in a 1:10 ratio was added to the milk and constantly agitated until milk pH = 4.5. Whole milk was mixed milk replacer (Herdfirst 20–20 AM Bov EZ, 20% CP, 20% crude fat, 0.15% crude fiber, Cargill, MN, USA) to correct the % total solid and deliver a mix with 13% total solids.

Upon arrival to the calf barn, farm personnel introduced all animals to the nipples, and training persisted for 2–3 days. The automated feeding system was cleaned daily, and all nipples were replaced weekly. All calves were offered pasteurized acidified milk at 25.5 °C *ad libitum* from arrival to the calf-raising facility until d 42. All pens had free access to fresh water at all times. On d 7, calves were offered *ad libitum* textured calf starter (Key Calf Starter, 18.10% CP, 4.00% crude fat, and 5.00% crude fiber, Keystone Mills, Romulus, NY) and at d 14 until d 56, to *ad libitum* hay in addition to the calf starter (21.01% CP, 2.99% crude fat, and 6.26% crude fiber, Keystone Mills, Romulus, NY, USA). Weaning of calves utilized a gradual step-down protocol initiated at d 42 with a 40% reduction of time for milk access till d 50, then a 90% reduction of time for milk access until d 56. All calves were disbudded around d 21 of age, with a butane cauterizer (Portasol, Carlow, Ireland). Prior to dehorning, a local anesthetic (2% lidocaine, Vetone, Boise, Idaho) combined with 0.5 mg/mL sedation (XylaMed, Vetone, Boise, ID, USA) was delivered to each cornual nerve. Each calf then received appropriate pain medication using transdermal flunixin (Banamine Transdermal, Merck, Madison, NJ, USA).

### 2.1. Data and Sample Collection

Days carried calf, parity, calving ease score (1–5), and dam parity were recorded for each calf enrolled in the study. Calves were weighed immediately after birth and weekly thereafter with Waypig 15 digital scale (Raytec LLC, Ephrata, PA, USA) until d 56. Withers heights were measured with a meter stick (NASCO, Fort Atkinson, WI, USA).

On d 2 to 7 after birth, at least 12 h after the second feeding, blood samples were collected from the jugular vein in all calves with a 22 G × 1” needle in a 10 mL red top tube (BD Vacutainer, Fisher Scientific, Waltham, MA, USA). A heparinized microhematocrit capillary tube (Kimble, Rockwood, TN, USA) was filled immediately from the red top tube, and the hematocrit was determined. The remaining sample was centrifuged at 3000× *g* at room temperature for 15 min for serum separation. The serum was evaluated using an optical Brix refractometer (Misco Palm Abbe #PA201, Cleveland, OH, USA), and the value was used to estimate each calf’s total serum protein. Failure passive transfer immunity was assumed at two different methods: (1) % Brix < 8.4 [23,24,25]; and (2) four different categories (Excellent [≥9.4]%, Fair [8.9–9.3]%, Good [8.1–8.8]%, and Poor [<8.1]%) [15,24].

Pneumonia and otitis were diagnosed, treated, and recorded by two trained farm personnel for all animals. All diagnoses and treatments of disease followed the standard operating procedures outlined in this commercial dairy. Otitis was defined as unilateral or bilateral ear droop, head tilt, cough, and or shallow breathing. Pneumonia was defined as shallow breathing, cough or noticeable effort to breathing, and rectal temperature > 39.7 °C. All disease events and treatments were recorded in DairyComp 305 (VAS, Tulare, CA, USA). Diarrhea events were not consistently recorded by farm personnel and were not used in this study. Once diarrhea was observed, calves with poor appetite and noticeable dehydration were fed 2 L of oral electrolytes (Land O’Lakes, Arden Hills, MN, USA) using an esophageal tube for 2 d. Calves that had no appetite and were severely dehydrated with sunken eyes were given 2 L Lactated Ringer’s Solution intravenously (Radix Labs, International Drive, Eau Claire, WI, USA). For respiratory and otitis, calves were treated once with tulathromycin 2.5 mg/kg subcutaneous (Draxxin, Zoetis, Parsippany, NJ, USA) and rechecked 7 d after the initial treatment. A second treatment using the same antimicrobial and same dose was given if the calf did not recover. Those that required a third treatment were given once tilmicosin 20 mg/kg subcutaneous (Micotil, Elanco, Greenfield, IN, USA).

### 2.2. Statistical Analysis

Prior to statistical analysis, one calf in the CON group was excluded from the study due to death in the first 48 h. The necropsy opportunity was not available. This resulted in a study population of n = 189. Chi-squared tests were performed using PROC FREQ of SAS (SAS 9.4) for differences in categorical variables such as dam parity, failure of passive transfer % Brix value < 8.4% [25], passive transfer failure four different categories (Excellent [≥9.4]%, Fair [8.9–9.3]%, Good [8.1–8.8]%, and Poor [<8.1]%) [15,24], ADG categories [26], hematocrit, and the number of antibiotic treatments. The correlation between hematocrit and serum % Brix was analyzed using Proc corr in SAS 9.4 and GraphPad Prism 9.2. One-way ANOVA was used for differences in continuous variables such as average weight at birth and serum % Brix. The weekly ADG and height were analyzed with repeated measures ANOVA using PROC Mixed in SAS 9.4. Five covariance structures were tested for the weekly ADG outcome (simple, compound symmetry, autoregressive order 1, Toeplitz, and unstructured), and the covariance structure with the lowest Akaike’s information criterion was selected. Treatment was included as a fixed effect, and the REPEATED statement was used for the time variable. Other plausible fixed effects terms (e.g., dam parity, days carried calf, and birth weight) were tested and not included in the final model if the *p*-value ≥ was 0.05. Block was included in this model as a random effect and to control for changes in management, weather, and other factors that we were not able to control. The treatment × time interaction was forced in the model. Other plausible interaction terms were tested and not included in the final model if the *p*-value is ≥ 0.05. Tukey’s post hoc test were used for multiple comparison correction of *p*-values for all pairwise comparisons of least square means. Normality and homoscedasticity of residuals were visually evaluated for the model fit. The average incidence of both diseases and the number of treatments between the control and treatment were compared using the one-way ANOVA method. Significance for all analyses was declared if *p* < 0.05. Univariable logistic regression models using PROC GLIMMIX were used to investigate potential associations between the treatment groups and respiratory diseases and otitis. Then, odds ratios were calculated for these associations.

## 3. Results

### 3.1. Description of Study Population and Treatment

The descriptive statistics of the study population are shown in Table 1. There was no difference in the % Brix of the first colostrum feed to both CON and COL (*p* = 0.43). Time interval from the first feeding of colostrum to the second feeding averaged (±SD) at 843 (±6) min [CON: 853 (614–1180), COL: 834 (665–1037); *p =* 0.12].

**Figure 1:**
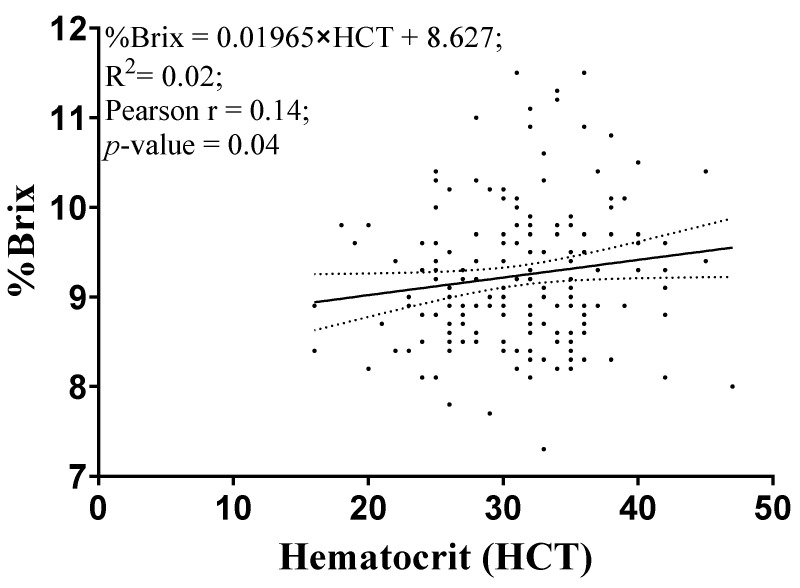
Linear regression analysis between the hematocrit concentration and serum % Brix. The solid line represents the linear regression, and the dotted lines represent the 95% CI.

The hematocrit (**HCT**) was similar in CON and COL groups (*p* = 0.31). However, serum % Brix concentration was greater in the COL group than CON (*p* < 0.01). We observed a very weak association between HCT and serum % Brix, Figure 1. 

There was no difference in the number of calves with passive transfer failure using the 8.4% Brix cut-off, 7 calves in COL, and 10 calves in CON (*p* = 0.40). Similarly, we did not observe a difference in the failure passive transfer immunity serum% Brix categories by treatment (*p* = 0.14; Table 2).

### 3.2. Average Daily Gain and Height

**Figure 2:**
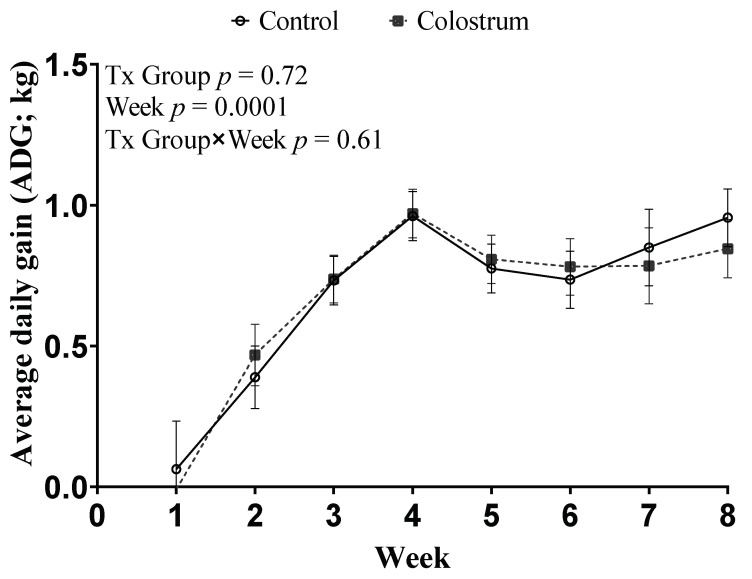
Least squares means of repeated measures ANOVA of ADG during the pre-weaning period. Treatments: control (CON) received 4 L of colostrum within 2 h of life followed with 3 L of acidified milk within 12–16 h later; Colostrum (COL) received 4 L of colostrum within 2 h of life followed with another 3 L of colostrum 12–16 h later. Error bars represent standard error. Enrollment block was included as a random effect.

Bodyweight increased over time from calving to weaning (*p* < 0.01) and were similar in both groups up to wk 8 (*p* = 0.70). The ADG during the entire pre-weaning period and weekly ADG between CON and COL are summarized in Figure 2.

**Figure 3:**
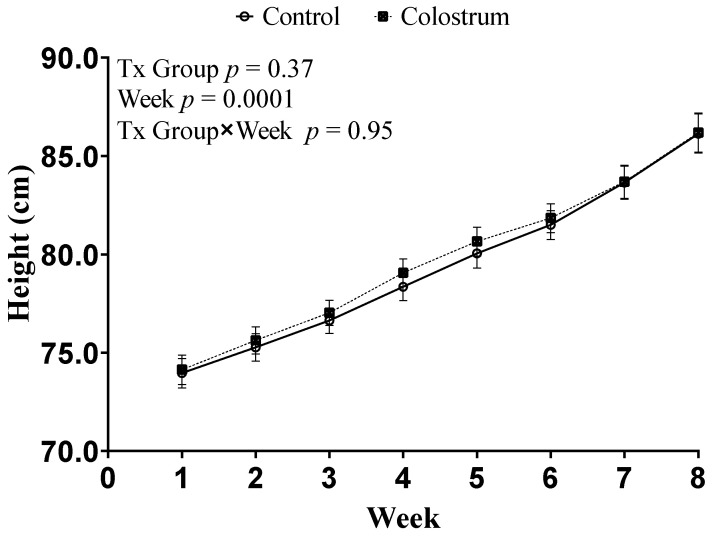
Least squares means of repeated measures ANOVA of withers height (cm) during the pre-weaning period. Treatments: control (CON) received 4 L of colostrum within 2 h of life followed with 3 L of acidified milk within 12–16 h later; Colostrum (COL) received 4 L of colostrum within 2 h of life followed with another 3 L of colostrum 12–16 h later. Error bars represent standard error. Enrollment block was included as a random effect.

We did not observe a difference in the ADG (95% CI) kg/d during the pre-weaning period between CON 0.68 (0.63–0.72) kg/d and COL 0.67 (0.63–0.71 kg/d; *p* = 0.72). In addition, there were no differences in ADG (*p* = 0.62) and height (Figure 3; *p* = 0.95) for treatment × Time interaction between CON and COL.

### 3.3. Health Status

In respiratory disease, 31.34% of the study population was treated for the respiratory disease at least once. Colostrum calves had a greater number of treatments for respiratory disease than CON at 56 and 38, respectively (*p =* 0.02). There was no significant difference in odds of developing a respiratory disease in COL than the CON calves [1.3 (95% CI; 0.65, 2.68; *p* = 0.43)]. Out of the total study population, 52.4% were treated for otitis at least once. The odds of developing otitis were not significantly different between CON and COL calves during the pre-weaning period [1.3 (95% CI; 0.72, 2.31; *p* = 0.38)]. The CON had a greater number of antibiotic treatments for otitis than COL at 141 and 116, respectively (*p* = 0.01). The overall antibiotic treatment was higher in CON than COL at 179 and 172, respectively (*p* = 0.001). There was no difference in mortality rate between the CON and COL groups during the first 8 wk of life, at 4.2% and 3.7% (*p* = 0.77), respectively.

## 4. Discussion

The study was performed to primarily investigate the effect of providing an additional feeding of good quality colostrum on the pre-weaning ADG in dairy Holstein heifers compared to those fed with acidified milk. Our study did not observe a difference in total body weight gain, ADG during the 56 d study period, weekly ADG, and withers height between the two groups.

Unique to our study situation, the farm at which the study was conducted experienced a malfunctioning feeding system. Cold unpasteurized acidified milk was delivered through the automated feeders to all pre-weaning heifers for approximately seven days. During this period and to reduce the effects of the malfunctioning automatic feeding system, all heifers were bottle-fed individually with pasteurized acidified milk by farm personnel. Despite great effort, the acute change in diet affected the enrolled heifers at the time, and our overall data starting from the ADG at week 4. The remaining of the data was also affected due to this cohort of calves. The ADG was compromised during this period equally for both the CON and COL. However, their total body weights continued to increase during this period. An increase in calf morbidity and mortality of calves was also equally observed for the CON and COL during this period.

Recent research in colostral bioactive factors has proposed prolonged feeding of colostrum may benefit the health of neonatal calves [27,28,29]. Explanations of these effects include better small intestinal absorption, as demonstrated by Hammon and Blum (1997) [27], where the xylose absorption test at d 5 was seen greater in calves that received 1 and 6 feedings of colostrum compared to those calves that received milk replacer only. In addition, consistent with other studies [5,30], significant greater villus heights, circumferences, and villus areas were morphologically observed as early as d 8 in those calves that have received colostrum than those that received milk replacer. Likely, these beneficial results are not only due to one bioactive factor but rather a synergistic effect and various intricate interactions at the molecular level that results in changes in the endocrine system and metabolic state. Nonetheless, the results of our study did not show an enhanced growth performance in calves that were fed with two feedings of colostrum. This may be in agreement with Hammon and Blum (1997) [27], where they did not observe a difference in the xylose absorption capacity at d 5 between the 1 or 6 feedings of colostrum treatment group. In addition, in the natural physiology of cattle, the colostral phase of mammary secretions may last up to 5–7 days post-partum [22]. Yet, the number of bioactive factors decrease significantly after 6 milkings or 3 days [3]. In our study, the potential beneficial effects of colostrum may also be masked as calves were provided with only one additional feeding 12–16 h post the first feeding. The duration of which colostrum was provided may not be sufficiently prolonged.

Moreover, providing a second feeding of colostrum is beneficial to achieve the new standards on calf- and herd-level passive immunity in dairy calves in the United States [24]. However, time and feeding techniques must be considered, especially in large dairy farms. In addition, calves are commonly reluctant to drink a second colostrum feeding from a bottle with a nipple shortly after the first feeding, <6 h. Therefore, esophageal tube feeding is an effective strategy for feeding a second dose of colostrum 12–16 h after the first colostrum feeding, obtaining greater serum % Brix in dairy calves.

Housing management may play a role in the effect of ADG in our study. Faber et al. (2005) [29] observed a higher pre-weaning ADG in Brown Swiss heifers that were provided with either 4 L of high-quality colostrum (50–140 mg/mL of IgG) at birth via bottle-feeding than those that were provided with 2 L. However, the animals were housed in individual pens. In our study, enrolled calves CON and COL were group-housed with other heifers that were not part of the study. Svensson et al., (2003) [31] reported that calves in large group-housing pens with an automatic feeding system had greater odds of developing severe or mild pneumonia than individually housed calves. This may affect ADG in calves.

The proportion of enrolled calves in our study in each pen ranged from 10–50% at any given period. Although numerous research has shown that grouped housed calves are associated with increased body weight gains than individually housed [32,33], group housing, along with other factors, are associated with higher calf morbidity [34,35]. However, these effects are group size-dependent [36]. Thus, the mass pen effect may have increased our calf morbidity and affected the ADG. Additionally, our study did not report on diarrhea and its effects on ADG.

It is important to note that the sample size calculation used in our study intended to find a biological difference in ADG. The current sample size may be insufficient to fully understand the effect of two colostrum feedings in calf disease incidence and mortality during the pre-weaning period. However, we found a reduction in the total number of antibiotic treatments during the pre-weaning period in the COL calves. This is in agreement with a recent study by Abuelo et al., 2021 [16]. However, the prevalence of respiratory disease reported by Abuelo et al., 2021 [16] is lower than the prevalence of respiratory disease observed in our study. This retrospective study analyzed data from individually housed calves [16].

Hemoconcentration has been associated with changes in total serum proteins in calves [37], and the HCT is a standard method to estimate hemoconcentration. We did not observe an association between serum % Brix and HCT in the current study. In our study, calves that were fed two feedings of colostrum approximately 12–16 h apart had greater serum % Brix than those calves with a follow-up the feeding of 3 L of acidified milk instead. There was no difference in calves with failure of passive transfer among the groups. The feeding method, by esophageal tubing, may not play a role in the observed differences in the IgG between the two groups. Desjardins-Morrissette et al., (2018) [38] have demonstrated that when colostrum is fed at higher quantities, 3 L compared to 1.5 L, abomasal emptying was not affected as the residual amount remained in the rumen is proportionally smaller. As the amount of colostrum or acidified milk fed to the calves was 4 L for the first feeding and 3 L for the second feeding in this study, it is unlikely slower abomasal emptying rate would have an effect on the observed results. In addition, Quigley et al., (2019) [39] observed that serum IgG was lower in calves that were fed 1.8 L of colostrum replacer followed by two feedings of 1.8 L of milk replacer within 24 h compared to those calves that were fed 1.8 L of pooled colostrum within the same time period. The study also attributed the low serum IgG in the group fed with milk replacer to dilution IgG in the colostrum replacer. A dilution effect may have minimal effect on our observed result, as milk curds in the abomasum are fully digested by 12–18 h, which is approximately the time of our second feeding [40,41,42].

Unlike other farm animal species where the cessation of absorption of macromolecules is greatly affected by the timing of first feeding [43,44], in bovine neonates, absorption is greatly reduced after 6–12 h of age [9], and cessation to absorb immunoglobulins is blocked entirely at 24–36 h of age [45,46]. Consistent with other studies, our result supports that the small intestine may continue to absorb IgG despite diminished absorption capacity, resulting in greater serum total proteins [9,47]. Although the incidence of evaluated disease events between the COL and CON was inconclusive, the total number of antibiotics used in the COL was lower, which may be attributed to the higher estimated serum IgG. Greater IgG concentration may not be sufficient to achieve optimal growth in dairy calves. Appropriate nutrition is critical during the pre-weaning period to allow the dairy calves to reach their maximum growth.

## 5. Conclusions

Providing calves with a second feeding of good quality colostrum 12–16 h after first feeding did not result in a more significant pre-weaning average daily gain compared with feeding those calves with milk in our current study. However, those calves that received two feedings of colostrum resulted in higher serum % Brix, required a lower number of antibiotic treatments for otitis. The benefits of feeding additional colostrum or transition milk high in bioactive factors may be seen with a more intensified feeding regime. Still, further studies are needed in group-housed calves.

## Figures and Tables

**Table 1:** Description of the study population and treatment are presented as total counts and 95% CI.

Measurement	^1^Treatments		
	Control	Colostrum	*p*-Value
Number of animals	94	95	-
Birth weight (kg)	38.0 (35.7–40.3)	38.8 (31.7–52.6)	0.19
Parity of dams	1.4 (1.2–1.7)	1.6 (1.3–1.8)	0.35
Days carried calf (days)	276.3 (267.1–285.4)	275.7 (266.3–285.0)	0.36
%Brix of first colostrum	25.2 (24.1–26.2)	24.9 (23.8–25.9)	0.43
%Brix of second colostrum	7.3 (6.5–8.2)	25.5 (24.7–26.4)	<0.01
Serum % Brix	9.2 (9.0–9.5)	9.7 (9.4–9.9)	<0.01

^1^Treatments: control (CON) received 4 L of colostrum within 2 h of life followed with 3 L of acidified milk within 12–16 h later; Colostrum (COL) received 4 L of colostrum within 2 h of life followed by another 3 L of colostrum 12–16 h later.

**Table 2:** Overall average daily gain (ADG) wk 1–8 and serum % Brix categories by treatment.

	^1^Treatments		
	Control	Colostrum	*p*-Value
**Categories serum % Brix**			
Excellent (≥9.4)%	32 (17.0%)	45 (23.8%)	0.14
Fair (8.9–9.3)%	28 (14.8%)	24 (12.7%)	
Good (8.1–8.8)%	29 (15.3%)	25 (13.3%)	
Poor (<8.1)%	5 (2.6%)	1 (0.5%)	
**Categories Average Daily Gain (ADG) WK 1–8**			
Excellent (ADG > 0.82) kg/d	12 (7.0%)	10 (5.8%)	0.57
Fair (ADG 0.64 to 0.82) kg/d	42 (24.4%)	36 (20.9%)	
Poor (ADG < 0.64) kg/d	33 (19.2%)	39 (22.7%)	

^1^Treatments: control (CON) received 4 L of colostrum within 2 h of life followed with 3 L of acidified milk within 12–16 h later; Colostrum (COL) received 4 L of colostrum within 2 h of life followed by another 3 L of colostrum 12–16 h later.

## References

[1] Larson B.L., Heary H.L., Devery J.E. (1980). Immunoglobulin Production and Transport by the Mammary Gland. J. Dairy Sci..

[2] Foley J.A., Otterby D.E. (1978). Availability, Storage, Treatment, Composition, and Feeding Value of Surplus Colostrum: A Review. J. Dairy Sci..

[3] Blum J.W., Hammon H.M. (2000). Bovine colostrum: More than just an immunoglobulin supplier. Schweiz. Arch. Tierheilkd..

[4] Mann S., Curone G., Chandler T.L., Moroni P., Cha J., Bhawal R., Zhang S. (2020). Heat treatment of bovine colostrum: I. Effects on bacterial and somatic cell counts, immunoglobulin, insulin, and IGF-I concentrations, as well as the colostrum proteome. J. Dairy Sci..

[5] Baumrucker C.R., Hadsell D.L., Blum J.W. (1994). Effects of dietary insulin-like growth factor I on growth and insulin-like growth factor receptors in neonatal calf intestine1. J. Anim. Sci..

[6] Playford R.J., Weiser M.J. (2021). Bovine Colostrum: Its Constituents and Uses. Nutrients.

[7] Roffler B., Fäh A., Sauter S.N., Hammon H.M., Gallmann P., Brem G., Blum J. (2003). Intestinal Morphology, Epithelial Cell Proliferation, and Absorptive Capacity in Neonatal Calves Fed Milk-Born Insulin-Like Growth Factor-I or a Colostrum Extract. J. Dairy Sci..

[8] Mann S., Curone G., Chandler T.L., Sipka A., Cha J., Bhawal R., Zhang S. (2020). Heat treatment of bovine colostrum: II. Effects on calf serum immunoglobulin, insulin, and IGF-I concentrations, and the serum proteome. J. Dairy Sci..

[9] Fischer A., Song Y., He Z., Haines D., Guan L., Steele M. (2018). Effect of delaying colostrum feeding on passive transfer and intestinal bacterial colonization in neonatal male Holstein calves. J. Dairy Sci..

[10] Hammon H.M., Blum J.W. (1998). Metabolic and Endocrine Traits of Neonatal Calves Are Influenced by Feeding Colostrum for Different Durations or Only Milk Replacer. J. Nutr..

[11] Hammon H.M., Zanker I.A., Blum J.W. (2000). Delayed Colostrum Feeding Affects IGF-I and Insulin Plasma Concentrations in Neonatal Calves. J. Dairy Sci..

[12] Hammon H.M., Steinhoff-Wagner J., Flor J., Schönhusen U., Metges C.C. (2013). Lactation biology symposium: Role of colostrum and colostrum components on glucose metabolism in neonatal calves 1,2. J. Anim. Sci..

[13] Ontsouka E.C., Albrecht C., Bruckmaier R.M. (2016). Invited review: Growth-promoting effects of colostrum in calves based on interaction with intestinal cell surface receptors and receptor-like transporters. J. Dairy Sci..

[14] Pyo J., Hare K., Pletts S., Inabu Y., Haines D., Sugino T., Guan L., Steele M. (2020). Feeding colostrum or a 1:1 colostrum:milk mixture for 3 days postnatal increases small intestinal development and minimally influences plasma glucagon-like peptide-2 and serum insulin-like growth factor-1 concentrations in Holstein bull calves. J. Dairy Sci..

[15] Godden S.M., Lombard J.E., Woolums A.R. (2019). Colostrum Management for Dairy Calves. Veter. Clin. N. Am. Food Anim. Pr..

[16] Abuelo A., Cullens F., Hanes A., Brester J.L. (2021). Impact of 2 Versus 1 Colostrum Meals on Failure of Transfer of Passive Immunity, Pre-Weaning Morbidity and Mortality, and Performance of Dairy Calves in a Large Dairy Herd. Animals.

[17] USDA APHIS|NAHMS Dairy Studies. https://www.aphis.usda.gov/aphis/ourfocus/animalhealth/monitoring-and-surveillance/nahms/NAHMS_Dairy_Studies.

[18] Virtala A.-M.K., Mechor G.D., Gröhn Y.T., Erb H.N. (1996). The Effect of Calfhood Diseases on Growth of Female Dairy Calves During the First 3 Months of Life in New York State. J. Dairy Sci..

[19] Shamay A., Werner D., Moallem U., Barash H., Bruckental I. (2005). Effect of Nursing Management and Skeletal Size at Weaning on Puberty, Skeletal Growth Rate, and Milk Production During First Lactation of Dairy Heifers. J. Dairy Sci..

[20] Soberon F., Raffrenato E., Everett R.W., Van Amburgh M.E. (2012). Preweaning milk replacer intake and effects on long-term productivity of dairy calves. J. Dairy Sci..

[21] Weaver D.M., Tyler J.W., VanMetre D.C., Hostetler D.E., Barrington G.M. (2000). Passive Transfer of Colostral Immunoglobulins in Calves. J. Veter. Intern. Med..

[22] McGrath B.A., Fox P.F., McSweeney P.L.H., Kelly A.L. (2016). Composition and properties of bovine colostrum: A review. Dairy Sci. Technol..

[23] Elsohaby I., McClure J.T., Keefe G.P. (2015). Evaluation of Digital and Optical Refractometers for Assessing Failure of Transfer of Passive Immunity in Dairy Calves. J. Veter. Intern. Med..

[24] Lombard J., Urie N.J., Garry F., Godden S., Quigley J.D., Earleywine T., McGuirk S., Moore D., Branan M., Chamorro M. (2020). Consensus recommendations on calf- and herd-level passive immunity in dairy calves in the United States. J. Dairy Sci..

[25] Deelen S.M., Ollivett T.L., Haines D.M., Leslie K.E. (2014). Evaluation of a Brix refractometer to estimate serum immunoglobulin G concentration in neonatal dairy calves. J. Dairy Sci..

[26] Shivley C.B., Lombard J.E., Urie N.J., Kopral C.A., Santin M., Earleywine T., Olson J., Garry F. (2018). Preweaned heifer management on US dairy operations: Part VI. Factors associated with average daily gain in preweaned dairy heifer calves. J. Dairy Sci..

[27] Hammon H., Blum J.W. (1997). Prolonged colostrum feeding enhances xylose absorption in neonatal calves. J. Anim. Sci..

[28] Burrin D.G., Stoll B., Guan X. (2003). Glucagon-like peptide 2 function in domestic animals. Domest. Anim. Endocrinol..

[29] Faber S.N., Faber N.E., McCauley T.C., Ax R.L. (2005). Case Study: Effects Of Colostrum Ingestion on Lactational Performance. Prof. Anim. Sci..

[30] Odle J., Zijlstra R.T., Donovan S.M. (1996). Intestinal effects of milkborne growth factors in neonates of agricultural importance. J. Anim. Sci..

[31] Svensson C., Lundborg K., Emanuelson U., Olsson S.-O. (2003). Morbidity in Swedish dairy calves from birth to 90 days of age and individual calf-level risk factors for infectious diseases. Prev. Veter. Med..

[32] Chua B., Coenen E., Van Delen J., Weary D. (2002). Effects of Pair Versus Individual Housing on the Behavior and Performance of Dairy Calves. J. Dairy Sci..

[33] Costa J.H.C., Meagher R.K., von Keyserlingk M.A.G., Weary D.M. (2015). Early pair housing increases solid feed intake and weight gains in dairy calves. J. Dairy Sci..

[34] Warnick V.D., Arave C.W., Mickelsen C.H. (1977). Effects of Group, Individual, and Isolated Rearing of Calves on Weight Gain and Behavior. J. Dairy Sci..

[35] Gulliksen S.M., Lie K.I., Sølverød L., Østerås O. (2008). Risk Factors Associated with Colostrum Quality in Norwegian Dairy Cows. J. Dairy Sci..

[36] Svensson C., Liberg P. (2006). The effect of group size on health and growth rate of Swedish dairy calves housed in pens with automatic milk-feeders. Prev. Veter. Med..

[37] Slanina L., Bomba A., Lehocký J., Paulík S., Polácek M., Batta G. (1984). Hemoconcentration in calves and its relation to the hematologic, protein, mineral and electrolyte profile. Veterinární Med..

[38] Desjardins-Morrissette M., van Niekerk J.K., Haines D., Sugino T., Oba M., Steele M.A. (2018). The effect of tube versus bottle feeding colostrum on immunoglobulin G absorption, abomasal emptying, and plasma hormone concentrations in newborn calves. J. Dairy Sci..

[39] Quigley J.D., Deikun L., Hill T.M., Suarez-Mena F.X., Dennis T.S., Hu W. (2019). Effects of colostrum and milk replacer feeding rates on intake, growth, and digestibility in calves. J. Dairy Sci..

[40] Burgstaller J., Wittek T., Smith G.W. (2017). Invited review: Abomasal emptying in calves and its potential influence on gastrointestinal disease. J. Dairy Sci..

[41] MacPherson J., Berends H., Leal L.N., Cant J.P., Martín-Tereso J., Steele M.A. (2016). Effect of plane of milk replacer intake and age on glucose and insulin kinetics and abomasal emptying in female Holstein Friesian dairy calves fed twice daily. J. Dairy Sci..

[42] Okada K., Kato J., Miyazaki T., Sato S., Yasuda J. (2010). The effect of frequent milk feeding on abomasal curd formation of Holstein calves. Anim. Sci. J..

[43] Lecce J.G., Morgan D.O. (1962). Effect of Dietary Regimen on Cessation of Intestinal Absorption of Large Molecules (Closure) in the Neonatal Pig and Lamb. J. Nutr..

[44] Lecce J.G. (1973). Effect of Dietary Regimen on Cessation of Uptake of Macromolecules by Piglet Intestinal Epithelium (Closure) and Transport to the Blood. J. Nutr..

[45] Stott G.H., Marx D.B., Menefee B.E., Nightengale G.T. (1979). Colostral Immunoglobulin Transfer in Calves II. The Rate of Absorption. J. Dairy Sci..

[46] Michanek P., Ventorp M., Weström B. (1989). Intestinal transmission of macromolecules in newborn dairy calves of different ages at first feeding. Res. Veter- Sci..

[47] Hare K.S., Pletts S., Pyo J., Haines D., Guan L.L., Steele M. (2020). Feeding colostrum or a 1:1 colostrum:whole milk mixture for 3 days after birth increases serum immunoglobulin G and apparent immunoglobulin G persistency in Holstein bulls. J. Dairy Sci..

